# Ultra-high-resolution and dual-energy computed tomography of carotid artery plaques differentiate symptomatic and asymptomatic patients by novel volumetric analysis

**DOI:** 10.1093/icvts/ivaf158

**Published:** 2025-06-30

**Authors:** Roland-Richard Macharzina, Simon Stemmler, Werner Vach, Thomas Winker, Jana Taron, Christopher L Schlett, Michael Weinbeck, Matthias Siepe, Martin Czerny, Fabian Bamberg, Thomas Zeller, Dirk Westermann, Martin Soschynski

**Affiliations:** Department of Cardiology and Angiology, University Heart Center Freiburg—Bad Krozingen, Bad Krozingen, Germany; Department of Cardiology and Angiology, University Heart Center Freiburg—Bad Krozingen, Bad Krozingen, Germany; Department of Anesthesiology, University Medical Center Hamburg—Eppendorf, Hamburg, Germany; Basel Academy for Quality and Research in Medicine, University of Basel, Basel, Switzerland; Institute of Neurology, University Heart Center Freiburg—Bad Krozingen, Bad Krozingen, Germany; Department of Diagnostic and Interventional Radiology, Medical Center—University of Freiburg, Freiburg, Germany; Department of Diagnostic and Interventional Radiology, Medical Center—University of Freiburg, Freiburg, Germany; Department of Cardiovascular Surgery, University Heart Center Freiburg—Bad Krozingen, Bad Krozingen, Germany; Department of Cardiac Surgery, University Hospital Bern, Bern, Switzerland; Department of Cardiovascular Surgery, University Heart Center Freiburg—Bad Krozingen, Bad Krozingen, Germany; Department of Diagnostic and Interventional Radiology, Medical Center—University of Freiburg, Freiburg, Germany; Department of Cardiology and Angiology, University Heart Center Freiburg—Bad Krozingen, Bad Krozingen, Germany; Department of Cardiology and Angiology, University Heart Center Freiburg—Bad Krozingen, Bad Krozingen, Germany; Department of Diagnostic and Interventional Radiology, Medical Center—University of Freiburg, Freiburg, Germany

**Keywords:** carotid, CT, plaque, stroke

## Abstract

**OBJECTIVES:**

The indication for carotid endarterectomy (CEA) mainly relies on the degree of stenosis and neurological symptoms. Plaque vulnerability has been associated with stroke risk, but identification on single-energy computed tomography (CT) has yielded heterogeneous results and is not routinely applied to clinical diagnostics. Hence, we intended to analyse CEA specimens for vulnerability features using dual-source CT and correlate these features with the presence of preprocedural symptoms.

**METHODS:**

CT was performed on 187 carotid plaque specimens using ultra-high-resolution and dual-energy imaging on a dual-source scanner. Plaques were separated into calcified versus non-calcified volumes and analysed concerning HU-density, calcifications and volumetric dual-energy indices (DEIs). Comparative statistical analysis of plaque characteristics was performed with respect to the presence of neurological symptoms.

**RESULTS:**

The degree of stenosis of symptomatic and asymptomatic plaques was indifferent (69.2 ± 12.3% vs 66.3 ± 13.7%). The highest diagnostic accuracies were obtained by the % calcified volume (AUC 0.63 (0.54–0.71)), average whole plaque HU (AUC 0.71 (0.64–0.79)), profound calcification (AUC 0.74 (0.66–0.81)), calcification spots <1 mm (AUC 0.71 (0.63–0.79)) and spotty calcification (AUC 0.74 (0.66–0.82)). The diagnostic accuracy for symptomatic plaques was insignificant using average non-calcified plaque HU (AUC 0.59 (0.48–0.65)), but significant using average non-calcified plaque DEI (AUC 0.66 (0.58–0.74)).

**CONCLUSIONS:**

Symptomatic plaques were identified best by measuring density of the whole, calcified or non-calcified plaque and via spotty, profoundly localized and less dense calcification. A volumetric DEI identifies symptomatic plaques with non-calcified plaque characteristics more accurately than single-energy CT. Future clinical studies are necessary to confirm these findings in patients.

## GRAPHICAL ABSTRACT

**Figure:**
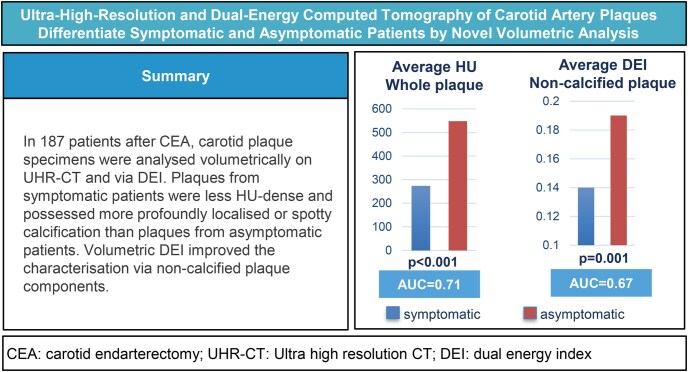

## INTRODUCTION

Carotid artery stenosis is responsible for 15–20% of ischaemic strokes and is highly relevant concerning overall morbidity and mortality [1]. Moreover, risk assessment from carotid stenosis is highly relevant for the prevention of perioperative stroke cardiac surgery [2]. The indication for revascularization mainly depends on the degree stenosis and neurological symptoms. Following guidelines, invasive therapy may be indicated at ≥60–70% stenosis for asymptomatic and ≥50% stenosis for symptomatic patients while considering clinical and imaging features as well as the surgical risk and the complication rate of the surgical centre [3–6]. However, in coronary artery disease (CAD), the additional risk of plaque composition is well known, and carotid plaques may similarly entail high risk of embolization at low-grade stenosis and vice versa [7–9]. Histologic plaque composition corresponds to plaque vulnerability; however, imaging these features has not entered routine clinical diagnostics [10]. While guidelines mention radiological features of vulnerable plaques (e.g. haemorrhage on magnetic resonance imaging), none are routinely considered as a main indication for carotid endarterectomy (CEA) [3, 4].

Attempts to establish Hounsfield unit (HU)-ranges from computed tomography (CT) that correspond to plaque components have, except for calcification, revealed great overlap [11, 12]. Macro-calcification appears to be inversely correlated with embolization risk, although contrary findings have been published [13–15]. There is contradicting evidence on localization of calcification [16–18]. Some argue that luminal calcification is prone to rupture and profound calcification acts as a barrier to inflammation [16, 17, 19], while others suggest that luminal non-calcified plaque with profound calcification might increase risk of rupture by inflammation of the fibrous cap [18, 20]. An inverse risk association of higher HU-density in calcification has been proposed [20].

No data on the risk association of metric HU-values including the non-calcified plaque have been published, especially not across the whole plaque volume. Most *in vivo* studies have analysed the plaque two-dimensionally or at multiple cross-sections with considerable distance between measurements, as volumetric quantifications are often complicated by luminal contrast agent and inadequate software capabilities. Hence, only allowing an approximation of a three-dimensional pathology, while *ex vivo* studies can provide volumetric data without major difficulty. Dual-energy CT was also used to accurately detect calcification and assess vulnerability features, nevertheless, no data exist on the association of volumetric dual-energy CT parameters with symptomatic plaques [21, 22].

The association of objective radiological plaque characteristics with symptoms remains unclear. This *ex vivo* study of CEA specimens aims to identify differences in plaques of symptomatic and asymptomatic patients using dual-energy CT for HU-density and volume of plaque components, as well as sub-classifications of calcification across the whole plaque volume. Moreover, this is the first study to analyse the association of a volumetric dual-energy index (DEI) with the symptomatic status. We hypothesized that volumetric HU-density and calcification sub-typing can identify plaques from symptomatic patients, and that DEI-images are more accurate at analyzing non-calcified plaques.

## METHODS

### Patient inclusion and specimen preparation

**Figure 1:**
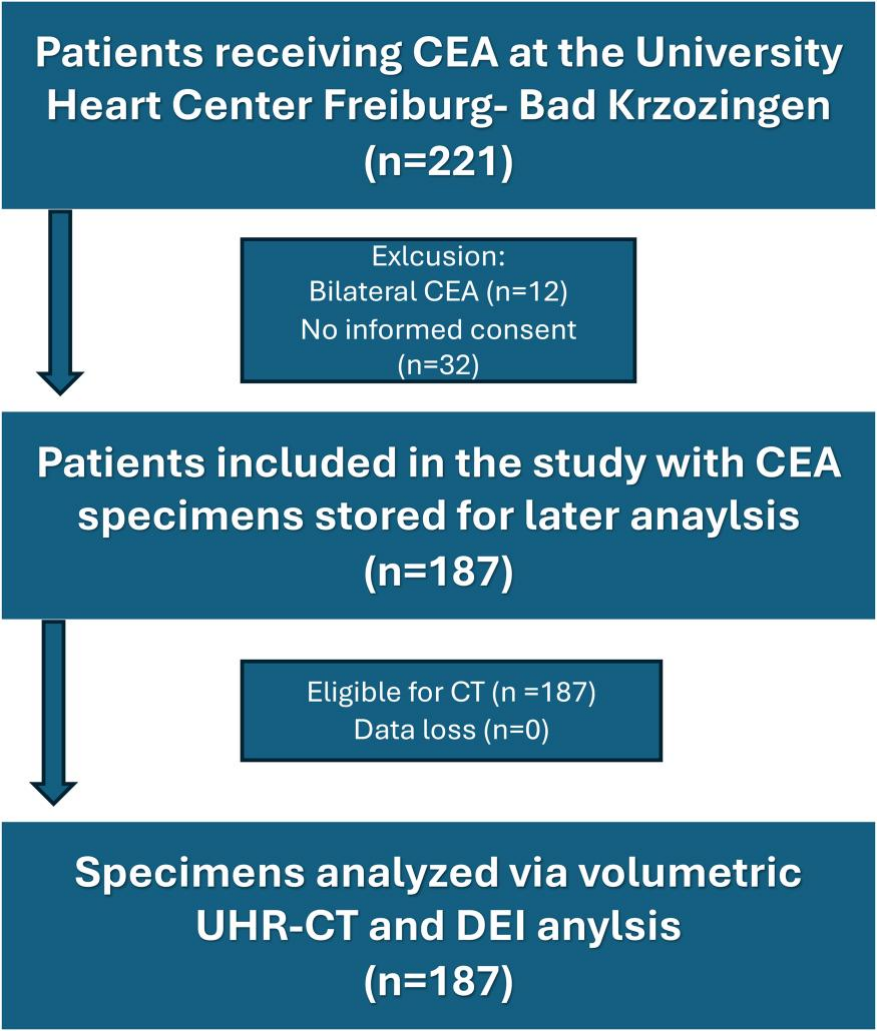
Flow chart. Flow chart of the study design (CEA: carotid endarterectomy; UHR-CT: ultra-high-resolution computed tomography; DEI: dual-energy index)

The study fulfilled ethical standards and rules of good clinical practice and was approved by the ethics committee of the Albert-Ludwigs-University Freiburg. Consecutive patients receiving CEA at the University Heart Center Freiburg—Bad Krozingen from 2012 to 2020 were enrolled (*n* = 221). Exclusion criteria were combined operations, cardiothoracic surgery within 6 months before CEA, bilateral CEA (*n* = 12) and missing informed consent (*n* = 32) (Fig. 1). Indication for revascularization was assumed in neurologically symptomatic patients with ≥50%-stenosis (i.e. suffering ipsilateral stroke or transient ischaemic attack (TIA) 6 months prior to the diagnosis) and for ≥70% asymptomatic stenosis and progressive disease while considering clinical circumstances [6]. The cohort of *n* = 187 patients was categorized into a neurologically symptomatic and an asymptomatic group as determined by a neurologist. All patients received a pre-operative neurology examination, routine clinical testing and pre-operative imaging using Doppler-Ultrasonography and CT-angiography. The North American Symptomatic Carotid Endarterectomy Trial (NASCET) degree of stenosis was quantified using validated grading criteria [23, 24]. CEA was performed by experienced vascular surgeons as eversion endarterectomy when feasible and under general anaesthesia. Near-infrared spectroscopy was used, and a shunt was placed after significant reduction in hemispheric oxygen saturation. Intraoperative angiography was performed to assess successful endarterectomy. The plaque was resected and specimens were rinsed, marked, fixed in pH-neutral 4%-formaldehyde solution and anonymously stored for later analysis.

### Computed tomography

**Figure 2:**
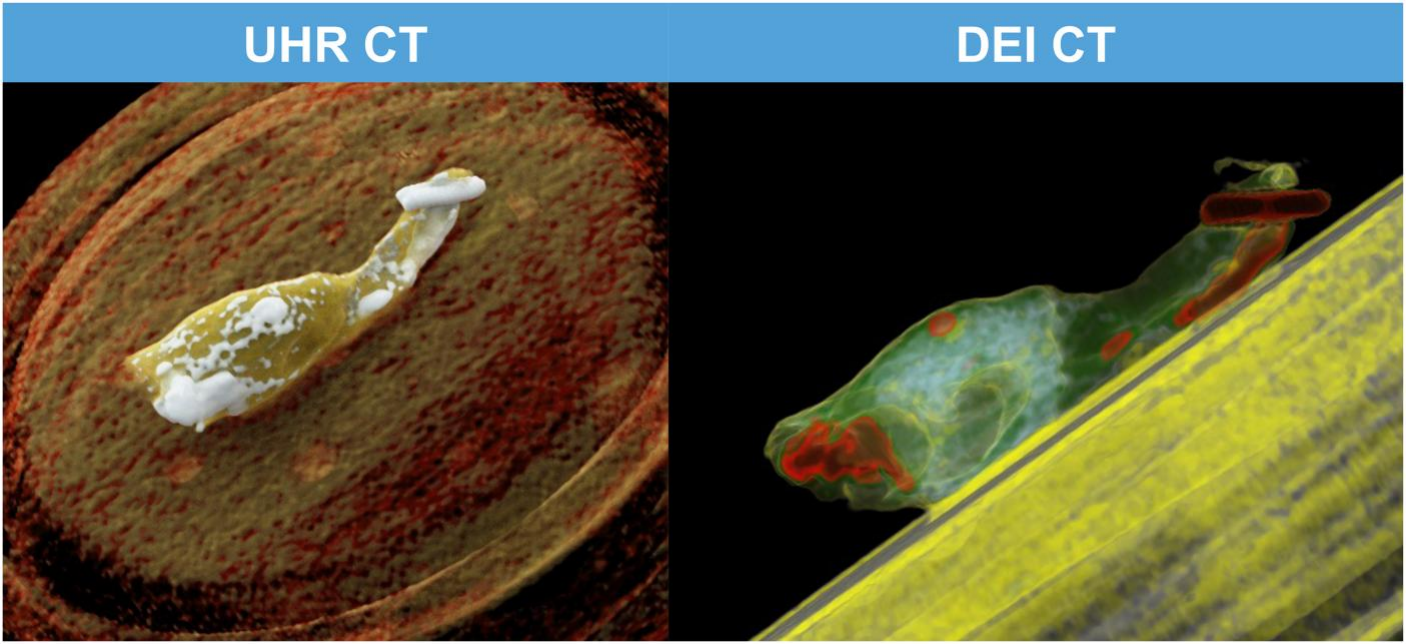
3D ultra-high-resolution computed tomography and dual energy index images

*Ex vivo* plaques were imaged using a third-generation dual-source CT (SOMATOM Force, Siemens Healthineers, Erlangen, Germany). Parameters of the first (single-energy) scan were: 120 kV, 500 mAs, 2 × 96 × 0.4 extra-ultra-high-resolution collimation, 0.5 pitch. Reconstruction parameters were: matrix-size 512 × 512 pixels, field-of-view 50.1 × 50.1 mm, slice thickness 0.4, voxel size 0.098 × 0.098 × 0.4 mm, Ur69-Kernel and 40L400W window. Parameters of the second (dual-energy) scan were: 70/Sn150 kV, 350/100 mAs. A 0.6 mm tin (Sn) filter was used for 150 kV images. Reconstruction parameters were: matrix-size 512 × 512 pixels, field-of-view 50.1 × 50.1 mm, slice thickness 0.5 mm, voxel size 0.098 × 0.098 × 0.5 mm and Qr54-Kernel. Multiplanar rendering was performed, and images were saved (Fig. 2).

### Imaging analysis

**Figure 3:**
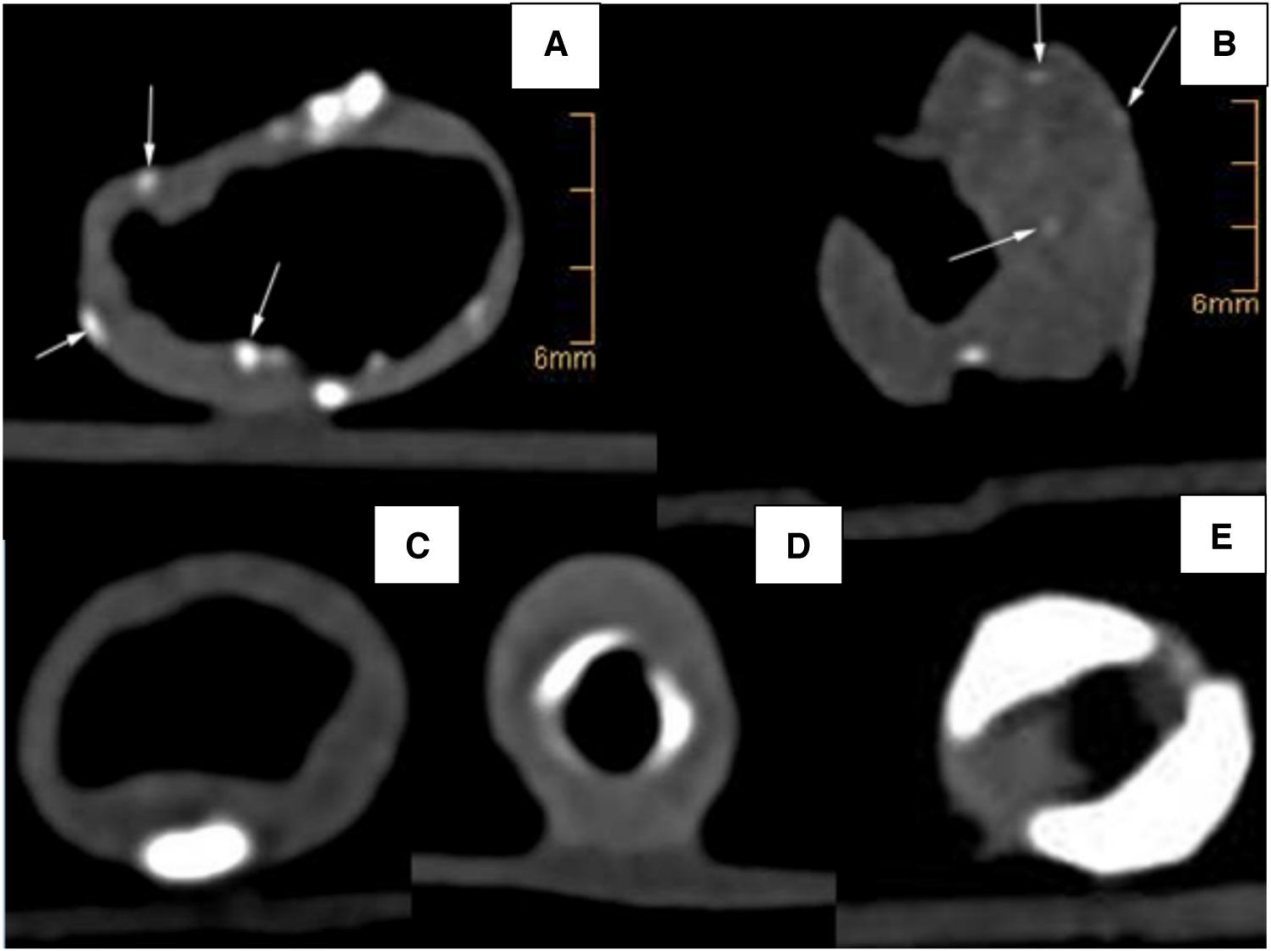
Calcification morphology and localization. (**A**) Spotty calcification—diffuse, dense, <1 mm (arrows). (**B**) Microcalcification—diffuse, barely measurable, density marginally above 130 HU (arrows). (**C**) Profound calcification. (**D**) Superficial calcification. (**E**) Superficial and profound calcification

Two experienced observers who were fully blinded to clinical data, the symptomatic status and other diagnostics analysed the images and disagreements were resolved in a consensus meeting. For qualitative data, inter-observer agreement was assessed. Qualitative analysis was performed with J-Vision (TIANI, Vienna, Austria) using plaque images derived from the 120 kV scan. Three to five characteristic cross-sections were analysed regarding circular calcification (0–4 = 0–4 quarters calcified, 5 = circular calcification), as suggested previously [25], superficial/luminal versus profound calcification, maximum/minimum density and quantified as shares of cross-sections with positive criterion or averages [25]. Calcification was defined as >130 HU, although average HUs of calcifications were markedly higher [26]. Spotty calcification has no clear definition and was considered diffuse calcification with a size of 1–5 mm, possibly linked to active inflammation [27, 28]. Microcalcification is presumably not visible on routine clinical imaging but recognizable on specialized imaging or histology [28, 29]. Calcification was evaluated as spotty (diffuse/non-confluent > 130 HU), microcalcified (spotty without measurable diameter and marginally > 130 HU) or macrocalcified (large, confluent, markedly > 130 HU) (Fig. 3). Calcification spots <1 mm and <3 mm were counted [28].

Quantitative volumetric analysis was performed using ImageJ (NIH, Bethesda, MD, USA). Voxels with −50 to 3000 HU were included to exclude titanium marking or air and to include all relevant plaque volume after consideration of the plaque’s maximum and minimum HU. For the volumetric dataset derived from the 120 kV scan, tables of mm³ volume per HU were generated and average, standard deviation and quartiles of HU-density were calculated for the whole, non-calcified (0–130 HU), and calcified plaque (>130 HU). Whole, non-calcified and calcified plaque volumes were calculated.

Dual-energy CT assesses the density properties of tissue in relation to photon energy by simultaneously acquiring images at two different tube voltages. A DEI is a simple method to quantify the density association across photon energy spectra [21].


(a) $$\mathrm{DEI}=\frac{\left({\text{HU}}_{\mathrm{Voxel}\;\;70\text{kV}}+100\right)-\left({\text{HU}}_{\mathrm{Voxel}\;\;\text{Sn}150\text{kV}}+100\right)}{\left({\text{HU}}_{\mathrm{Voxel}\;\;70\text{kV}}+100\right)+\left({\text{HU}}_{\mathrm{Voxel}\;\;\text{Sn}150\text{kV}}+100\right)}$$

This DEI (modified from Barreto *et al.*, with differing HU correction due to the preceding air exclusion) differentiates lipid-rich necrotic from fibrous plaque tissue with low overlap [21, 30].

Dual-energy data were transformed to DEI images using formula (a) applied to all voxels with −100 to 3000 HU, resulting in an image with voxels possessing a DEI instead of HU (Supplementary Material, Fig. S1). The differing HU range was selected according to maximum and minimum HU measurements of the plaque in 70 and Sn150 kV images to avoid plaque material exclusion. Tables of DEI values with corresponding number of voxels were generated and calculations were performed identically to the volumetric analysis of 120 kV-HU images.

### Statistics

SPSS Version-25 (IBM, Armonk, NY, USA) was used for statistical analysis. Metric data are presented as average ± standard deviation or point estimator (95% confidence interval). Patient and plaque characteristics were compared using an independent *t*-test for metric and Chi-square test for binary data. Statistical significance was assumed at *P* ≤ 0.05. Significant differences between the groups were further analysed via multiple logistic regression as odds ratios (OR) adjusted to the range of values, areas under the receiver operator characteristics (ROC) curve (AUC), sensitivity and specificity. Diagnostic cuts were calculated for selected criteria optimized for accuracy, sensitivity or specificity. Volumes derived from DEI and HU-values were compared using Pearson’s correlation coefficient (*r*) and Lin’s concordance correlation coefficient (CCC). Inter-observer agreement was quantified using Cohen’s Kappa.

## RESULTS

### Patient characteristics

Grouped patient characteristics are outlined in Table 1. Cohort risk factors are representative of cardiovascular centres [31]. Neurological events in the symptomatic group (*n* = 73) were: stroke (23%), TIA (41%) and amaurosis fugax (36%). There was no significant difference between the groups except for higher prevalence of CAD in asymptomatic patients, which became insignificant when patients receiving CEA for future CAD bypass surgery were excluded (44% vs 47%, *P* = 0.08).

**Table 1:** Risk factors and cohort characteristics of *n* = 187 patients

Parameters	All *n* = 187	Symptomatic *n* = 73	Asymptomatic *n* = 114	*P*
	Avg ± SD or *n* (%)	Avg ± SD or *n* (%)	Avg ± SD or *n* (%)	
NASCET stenosis (%)	67.4 ± 13.2	69.2 ± 1.3	66.3 ± 13.7	>0.1
Male gender	142 (76)	58 (79)	84 (74)	>0.1
Age (years)	70.4 ± 8.4	69.9 ± 10.1	70.7 ± 7.1	>0.1
Coronary artery disease (CAD)	105 (56)	32 (44)	73 (64)	0.007
Without bypass surgery patients	86 (51)	32 (44)	54 (47)	0.08
Previous myocardial infarction	30 (16)	8 (11)	22 (19)	>0.1
Periphery artery disease (PAD)	57 (30)	18 (25)	39 (34)	>0.1
Previous stroke or transitory ischaemic attack (TIA)	42 (22)	12 (16)	30 (26)	>0.1
Hypertension	175 (94)	68 (93)	107 (94)	>0.1
Hypercholesterolemia	177 (95)	67 (92)	110 (96)	>0.1
Diabetes mellitus II	50 (27)	16 (22)	34 (30)	>0.1
Smoking	52 (28)	22 (30)	30 (26)	>0.1
Contralateral carotid occlusion	5 (3)	1 (1)	4 (4)	>0.1
Renal insufficiency	36 (19)	16 (22)	20 (18)	>0.1
Atrial fibrillation	27 (14)	12 (16)	15 (13)	>0.1

### Plaque morphology on CT

Multiple morphologic features which were characteristic for symptomatic plaques are outlined in Table 2. Total plaque volume was indifferently distributed (600 ± 317 mm³ symptomatic vs 520 ± 283 mm³ asymptomatic, *P* = 0.08), while symptomatic plaques had higher non-calcified plaque volumes (73 ± 19% vs 65 ± 21%, *P* = 0.01) and were diametrically less calcified. HU-densities of the whole, calcified and non-calcified plaque volume were lower in symptomatic plaques. The whole plaque displayed significantly decreased HU-values at the average (274 ± 293 HU vs 548 ± 436 HU, *P* < 0.001) and 75th-volume quantile (421 ± 541 HU vs 631 ± 585 HU, *P* = 0.02), while only significant in the calcified plaque at the average (808 ± 303 HU vs 937 ± 252 HU, *P* = 0.002), maximum (1513 ± 753 HU vs 1994 ± 1024 HU, *P* < 0.001) and 25th/75th-quantile, and only significant in the non-calcified plaque at the minimum (−15 ± 15 HU vs −8 ± 24 HU, *P* = 0.04) and the average quantile as a trend (57.2 ± 6.8 vs 58.8 ± 6.2, *P* = 0.099). There was no inter-observer variability for the volumetric analysis due to fully automated measurement algorithms.

**Table 2:** Morphological comparison of symptomatic and asymptomatic plaques using ultra-high-resolution (HU) and dual-energy (DEI) computed tomography

Parameters	All *n* = 187	Symptomatic *n* = 73	Asymptomatic *n* = 114	*P*
	Avg ± SD or *n* (%)	Avg ± SD or *n* (%)	Avg ± SD or *n* (%)	
Total volume (mm³)	551 ± 296	600 ± 317	520 ± 283	0.08
Non-calcified plaque (%)	68 ± 20	73 ± 19	65 ± 21	0.01
Whole plaque average (HU)	441 ± 380	274 ± 293	548 ± 436	<0.001
Average (DEI)^a^	0.3 ± 0.12	0.26 ± 0.11	0.33 ± 0.13	<0.001
Calcified plaque average (HU)	887 ± 272	808 ± 303	937 ± 252	0.002
Average (DEI)	0.44 ± 0.07	0.41 ± 0.1	0.46 ± 0.05	<0.001
Maximum (HU)	1806 ± 918	1513 ± 753	1994 ± 1024	<0.001
Non-calcified plaque average (HU)	58.2 ± 6.4	57.2 ± 6.8	58.8 ± 6.2	0.099
Average (DEI)	0.21 ± 0.09	0.18 ± 0.08	0.23 ± 0.1	0.001
Minimum (HU)	−11 ± 20	−15 ± 15	−8 ± 24	0.04
Calcification spots <1 mm (*n*)	2.9 ± 3.3	4.3 ± 4.3	2 ± 2.6	<0.001
Circle index average	1.7 ± 1.2	1.3 ± 1.1	2 ± 1.2	<0.001
Spotty (cross-section shares)	0.39 ± 0.5	0.57 ± 0.38	0.27 ± 0.57	<0.001
Profound localization only (share)	0.39 ± 0.35	0.6 ± 0.38	0.25 ± 0.33	<0.001
Spotty calcification (0/1)	63 (34)	35 (48)	28 (25)	<0.001
Microcalcification (0/1)	8 (4)	7 (10)	1 (1)	0.003

a Dual Energy Index.

Symptomatic plaques displayed a higher portion of cross-sections with profound calcification only (60 ± 38% vs 38 ± 25%, *P* < 0.001) and spotty calcification (57 ± 38% vs 27 ± 57%, *P* < 0.001). Metric analysis of spotty calcification as the number of spots resulted in similar differences (<3 mm: 5.1 ± 4.9 vs 2.6 ± 2.8, *P* < 0.001; <1 mm: 4.3 ± 4.3 vs 2 ± 2.6, *P* < 0.001).

Calcification extended to fewer quarters in the circle-index of symptomatic plaques (1.3 ± 1.1 vs 2 ± 1.2, *P* < 0.001). Binary criteria significantly more frequent in symptomatic plaques were: spotty calcification (48% vs 25%, *P* < 0.001) and microcalcification (10% vs 1%, *P* = 0.003).

**Figure 4:**
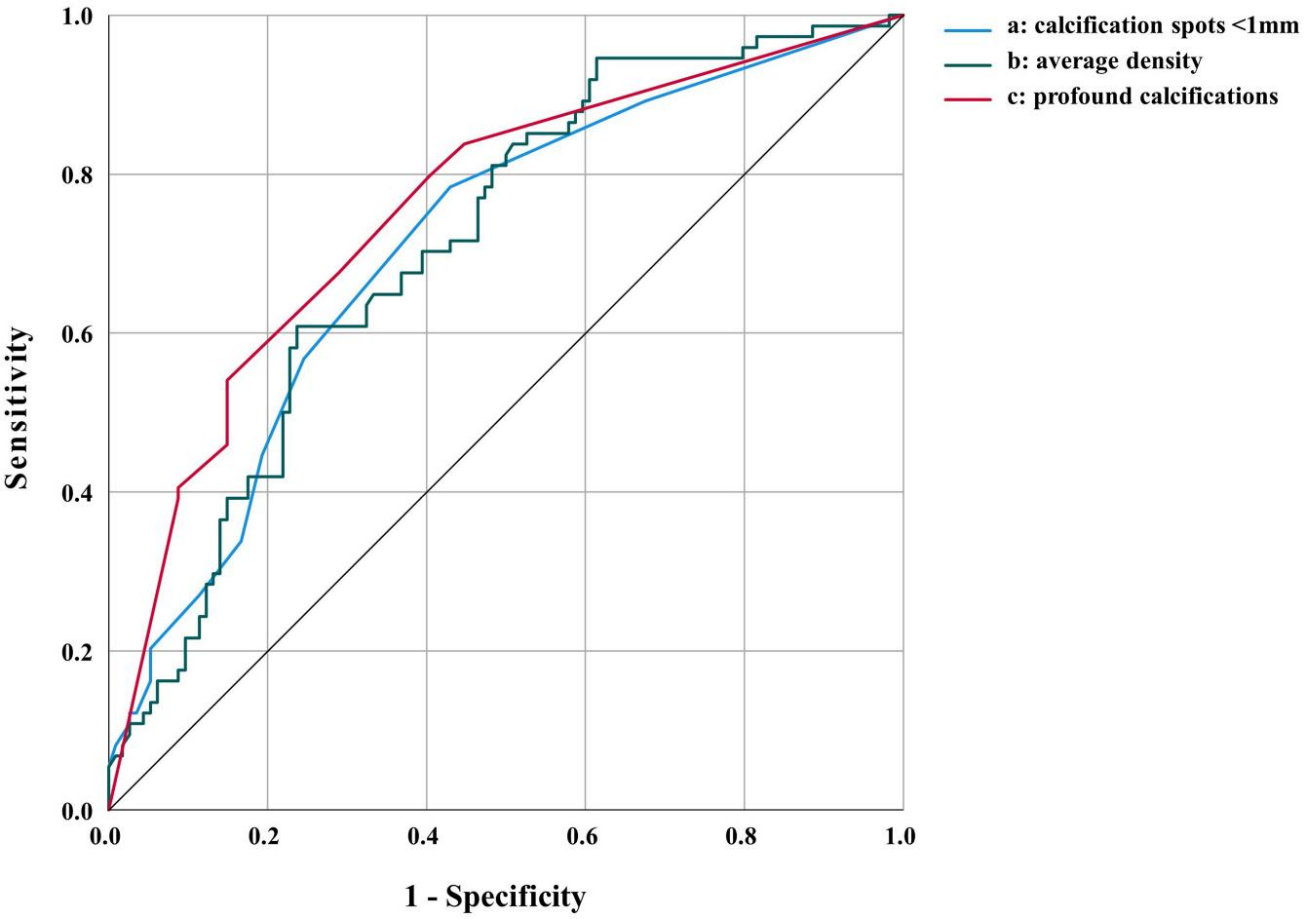
ROC curves of selected carotid plaque vulnerability criteria

ORs, AUCs and sensitivity/specificity for statistically different parameters across groups are shown in Table 3. High AUCs were achieved using the HU-density (whole plaque average HU: OR = 0.8 (0.72–0.89), AUC 0.71 (0.64–0.79), cut: <194 HU, 60.6% (53.6–67.6) sensitivity and 76.3% (70.2–82.4) specificity), calcification localization (shares with profound calcification only: OR = 1.3 (1.2–1.5), AUC = 0.74 (0.66–0.81), cut not calculable) and calcification morphology (shares with spotty calcified cross-sections: OR = 1.2 (1.1–1.3), AUC = 0.74 (0.66–0.82), cut not calculable) as well as metric spotty calcification (number of spots <1 mm: OR = 1.2 (1.1–1.4), AUC 0.71 (0.63–0.79), cut >2, 78.8% (72.9–84.7) sensitivity and 54.5% (47.7–61.6) specificity) (Fig. 4). High accuracies were also achieved by assessing spotty calcification as a binary criterion across the whole volume at 59.3% (52.3–66.3) sensitivity and 72.8% (66.2–79.2) specificity. Notably, the presence of microcalcification was highly specific for symptomatic plaques resulting in only one false positive diagnosis (99% (98–100) specificity and 11.9% (7.–-16.5) sensitivity). Inter-observer agreement for visual assessments were at least substantial (Cohen’s Kappa: 0.73 (spotty calcification) to 0.94 (profound calcification only)).

**Table 3:** Plaque parameter associations with symptomatic status using ultra-high-resolution and dual-energy computed tomography (*n* = 187)

Parameters	OR (CI)	*P*	AUC (CI)	*P*	Cut^a^	Sensitivity (%)	Specificity (%)
Non-calcified volume (%)	1.27*^b^ (0.72–0.89)	0.004	0.62 (0.53–0.71)	0.01	75.4	56.1 (49.0–63.2)	66.4 (59.6–73.2)
Whole plaque average HU	0.8** (0.72–0.89)	<0.001	0.71 (0.64–0.79)	<0.001	194	60.6 (53.6–67.6)	76.3 (70.2–82.4)
Whole plaque average DEI^c^	*0.6 (0.54–0.83)	<0.001	0.67 (0.59–0.75)	<0.001	0.27	59.4 (52.4–66.4)	69.4 (62.8–76.0)
Maximum HU	0.93** (0.9–0.97)	0.001	0.66 (0.58–0.75)	<0.001	1879	58.3 (51.2–65.4)	60.9 (53.9–67.9)
Calcified average DEI	*0.44 (0.27–0.71)	0.001	0.63 (0.54–0.71)	<0.001	0.43	36.2 (29.3–43.1)	81.1 (75.5–86.7)
Non-calcified average HU	0.68* (0.43–1.1)	0.1	0.59 (0.48–0.65)	0.12	56.9	64.9 (56.9–70.9)	46.6 (39.6–53.5)
Non-calcified average DEI	*0.56 (0.38–0.81)	0.002	0.66 (0.58–0.74)	<0.001	0.2	60.9 (53.9–67.9)	67.6 (60.9–74.3)
Calcification spots <1 mm	1.23 (1.1–1.4)	<0.001	0.71 (0.63–0.79)	<0.001	2	78.8 (72.9–84.7)	54.5 (47.7–61.6)
Spotty calcification (share^d^)	*1.19 (1.1–1.3)	<0.001	0.74 (0.66–0.82)	<0.001	n.c^e^	n.c.	n.c.
Profound calcification only (share)	*1.28 (1.2–1.5)	<0.001	0.74 (0.66–0.81)	<0.001	n.c.	n.c.	n.c.
Spotty calcification	3.9 (2.0–7.7)	<0.001	n.c.	n.c.	n.c.	59.3 (52.3–66.3)	72.8 (66.4–79.2)
Microcalcification	12.4 (1.5–102.7)	0.02	n.c.	n.c.	n.c.	11.9 (7.3–16.5)	99 (98.0–100.0)

a Maximum accuracy.

b *OR (increase by 0.1), OR* (increase by 10)….

c Dual-energy index.

d Shares of 3–5 cross-sections.

e Not calculable.

### Plaque morphology on dual-energy CT

Differences in volumetric DEI of the whole, calcified and non-calcified plaque are shown in Table 2. The average DEI was significantly lower in symptomatic plaques concerning the whole, calcified and non-calcified plaque (0.26 ± 0.11 vs 0.33 ± 0.13, *P* < 0.001; 0.41 ± 0.1 vs 0.46 ± 0.05; 0.18 ± 0.08 vs 0.23 ± 0.1, *P* = 0.001). There was very high correlation of plaque volume between volumetric calculations via DEI (70/Sn150 kV) and HU-images (120 kV) (565 ± 240 mm³ (DEI) and 551 ± 298 mm³ (HU), *r* = 0.88 (0.85–0.91), CCC = 0.87 (0.84–0.90)).

ORs, AUCs and sensitivity/specificity for statistically different parameters across the groups are shown in Table 3. The highest accuracy was achieved using the DEI of the 25th whole plaque volume quantile (OR = 0.67 (0.54–0.83), AUC 0.68 (0.6–0.76), cut <0.1DEI, 55.1% (38.0–62.2) sensitivity, 72.1% (65.7–78.5) specificity). Notably, the non-calcified plaque average DEI also achieved a high accuracy (OR = 0.56 (0.38–0.81), AUC = 0.66 (0.58–0.74), cut <0.2DEI, 60.9% (53.9–67.9) sensitivity, 67.6% (60.9–74.3) specificity) and was more accurate than the statistically insignificant accuracy of the non-calcified plaque HU-average, although the difference was not statistically significant (AUC = 0.66 (0.58–0.74) vs 0.59 (0.48–0.65), *P* = 0.24). The sensitivity-weighted cut with the highest specificity was average DEI of the whole plaque <0.41DEI at 90% (85.7–94.3) sensitivity and 29.7% (23.2–36.2) specificity. The specificity-weighted cut with the highest sensitivity was whole plaque DEI of the 25th volume quantile <0.04DEI at 91% (86.9–95.1) specificity and 37.5% (21.1–33.9) sensitivity. A multiple logistic regression including the degree of stenosis and cardiovascular risk factors as co-variables did not alter OR in terms of magnitude or significance in a relevant manner for any parameter.

## DISCUSSION

In this *ex vivo* imaging study, symptomatic plaques were discriminated from asymptomatic plaques with various morphological imaging-criteria, independent of the degree of stenosis, using high-resolution dual-source CT technology on CEA plaque specimens. This is the first study to associate low HU values, low DEI of the non-calcified plaque, metric spotty calcification and microcalcification with symptomatic carotid plaques volumetrically and supports various previously described risk associations on a quantitative volumetric basis. Several parameters demonstrated potential to discriminate symptomatic from asymptomatic plaques by demonstrating statistically significant differences and AUC values in the magnitude of 0.7. However, the clinical application remains to be determined by future *in vivo* studies.

Separating plaques into non-calcified and calcified portions at 130 HU appears feasible and provides the possibility to calculate proportions from volumetric measurements, especially considering the strength of CT in assessing calcified proportions more accurately than other imaging modalities. Hence, non-calcified plaque volume can be used as a simple metric tool to assess the risk of carotid plaques, as an increase of 10% in non-calcified volume elevates the probability of a plaque being symptomatic by 22% and could provide a qualitative risk impression. Plaque density can be objectively quantified across all plaque voxels, subdivided into whole, calcified and non-calcified plaque and described as average or quartile HU. All plaques show the association of lower density with neurological symptoms. Pathophysiological vulnerability criteria such as a lipid-rich necrotic core, haemorrhage and lipid-filled macrophages might lower the HU density additively from non-vulnerable fibrous tissue [11, 12, 21]. This might explain why investigations focusing on single vulnerability criteria have not yielded similar results [11, 12, 21]. Moreover, less dense calcifications might be an earlier stage of plaque maturation, still associated with certain levels of inflammation that have caused the calcification initially [32]. The high accuracy of the average whole plaque HU can be calculated semi-automatically without observer bias and detected without contrast agent, potentially decreasing side effects in an *in vivo* setting when the quantification of the % stenosis is not necessary and the lumen and surrounding tissue can be differentiated from plaque tissue. Still, the applicability of this method *in vivo* needs to be further assessed and translated to contrast-enhanced images with sufficient image post-processing. Since the *in vivo* applicability of such sophisticated analyses appears cumbersome with the current technical standard, an automatic approach using software that might delineate plaque volume and calculate values appears highly relevant for future studies. An increase in average whole plaque density by 100 HU can be interpreted as decreasing the probability of being symptomatic by 20%, hence a plaque with an average HU of 50 would be six times more likely to be symptomatic than one with an average HU of 850. The maximum HU density as a surrogate parameter of *old*, inactive calcification might be considered as a confirmatory criterion with high specificity and acceptable sensitivity, although it might be susceptible to artifacts [20].

The association of lower HU with the symptomatic status in the non-calcified plaque was weaker, but improvable by DEI measurements. This improvement might be due to enhanced differentiation of lipids from fibrous tissue with dual-energy imaging and should be investigated further *in vivo* via the volumetric DEI of the non-calcified plaque [30]. However, non-calcified plaque remains hard to image on CT, favoring magnetic resonance imaging and ultrasound are and necessitating risk-benefit analysis between complex CT measurements and other imaging modalities, potentially in a multimodal analysis. No relevant exclusion of plaque material from DEI calculation was detectable, as there was high correlation between mm³ voxels calculated via HU and DEI images, suggesting high internal validity. Lowered whole plaque DEI of the 25th volume percentile provides a sensitivity-weighted cut maintaining the highest specificity, although inacceptable for singular diagnostics. The differences in AUC values of DEI and HU measurements were too small to allow firm conclusions about superiority of one parameter.

Calcifications can be characterized on CT according to size, distribution, morphology and density. The inverse risk association of large, dense calcification described previously was confirmed in our study [13, 15]. Suggestions for direct risk associations might therefore be due to vulnerable subtypes of calcification or a comparison of calcified versus physiological carotids instead of non-calcified plaque [14]. Visually classifying plaques as spotty is simple and yields high accuracy, however, a quantitative method of counting calcification spots <1 mm appears to be less prone to inter-observer variability and results in an even higher accuracy. Nevertheless, *in vivo* resolution limitations and efficiency concerns must be considered. Still, this counting method could guide the diagnosis as every spot detected <1 mm increases the risk of being symptomatic by 23%. Detectability of microcalcification on modern *in vivo* CT imaging is unclear, but the presence of diffuse calcification marginally >130 HU may provide an extraordinarily specific criterion, although the low prevalence especially at lower resolution must be considered [27, 29]. Calcification localized profoundly instead of superficially (at the lumen) was also associated with symptomatic plaques. Although the presence of only profound calcification in shares of few cross-sections cannot provide diagnostic cuts, it may influence the patient’s risk assessment, as active, inflammatory processes might be localized outside calcified areas and make the plaque prone to rupture via lytic enzymes near the fibrous cap [18, 33]. Contrary evidence might result from the inclusion of co-presence with luminal protrusion as a risk factor despite luminal calcification and should be considered when attributing low risk to a luminally calcified plaque [16, 17]. A lowered circle index defined by Katano *et al.* displayed stronger associations with symptoms than calcification volume alone, suggesting an additional protective effect of circular calcification as a useful, easily visible criterion on CT [26]. Aiming at clinical applicability, strategies to bridge the gap between these *ex vivo* data and patient scans may include more focused scan fields via ultrasound marking to decrease radiation exposure and (semi)-automated software for volumetric plaque analysis via HU-cut off criteria in future clinical trials.

### Limitations

Translation of *ex vivo* criteria necessitates further *in vivo* validation, especially considering lower contrast of plaque to surrounding tissue than to air and difficulties of delineating the lumen without contrast agent. Nonetheless, improved resolution might also be achieved *in vivo*, potentially by selecting a smaller scan field under ultrasound-guided localization marking, or with photon-counting CT scanners offering high resolution and multi-energy scans simultaneously.

Given the *ex vivo* setting, only patients with the indication for CEA were included, providing limited evidence for potentially vulnerable plaques with <50% stenosis [9, 33].

Notably, past studies have shown similar associations irrespective of using a retrospective symptomatic status or the prospective occurrence of symptoms as the main endpoint [34].

This study does not include a histological reference to validate imaging features. This study did not examine relationships to other imaging modalities, needing further evaluation. Although the complexity of the analysis might be software driven, it still appears not clinically feasible without further research.

CT remains the best tool for assessing calcification, but a multimodal approach appears important considering strengths of ultrasound concerning economical application and resolution and those of MRI for detecting haemorrhages or fatty component.

## CONCLUSION

Multiple criteria can be identified using ultra-high-resolution *ex vivo* dual-source CT to discriminate symptomatic from asymptomatic plaques, however further research on patients is necessary to confirm these findings. Considering the whole plaque volumetrically concerning calcification and HU-density aids to assess the plaque’s symptomatic status, while the non-calcified plaque portion might provide better discrimination when regarded as DEI instead of HU. Moreover, sub-classifying calcification as profound only, spotty, non-circular, less dense, microcalcified and quantifying calcification spots <1 mm might provide further evidence for a vulnerable, symptomatic plaque. The assessment of metric plaque vulnerability associations appears as a promising tool to improve the risk–benefit evaluation before revascularization, while still taking the currently unclear *in vivo* applicability, degree of stenosis and other imaging modalities as well as the patient’s clinical situation into account.

## Supplementary Material

ivaf158_Supplementary_Data

## Data Availability

The data underlying this article are summarized in the article—detailed data (patient by patient) are available upon specific request to the corresponding author.

## ABBREVIATIONS

CAD: Coronary artery disease
CCC: Concordance correlation coefficient
CEA: Carotid endarterectomy
CT: Computed tomography
DE: Dual-energy
DEGUM: German Society for ultrasound in medicine
DEI: Dual-energy index
HU: Hounsfield units
NASCET: North American Symptomatic Carotid Endarterectomy Trial
OR: Odds ratios
TIA: Transitory ischemic attack
UHR: Ultra-high-resolution
