# Supplementary material for: Ultra-high-resolution and dual-energy computed tomography of carotid artery plaques differentiate symptomatic and asymptomatic patients by novel volumetric analysis
Source: Interdiscip Cardiovasc Thorac Surg. 2025 Jun 30;40(7):ivaf158. doi: 10.1093/icvts/ivaf158 (PMC12270255; doi:10.1093/icvts/ivaf158)
Supplement: ivaf158_Supplementary_Data [file ivaf158_supplementary_data.zip › Supplement.docx]

## **Supplement Fig. 1**


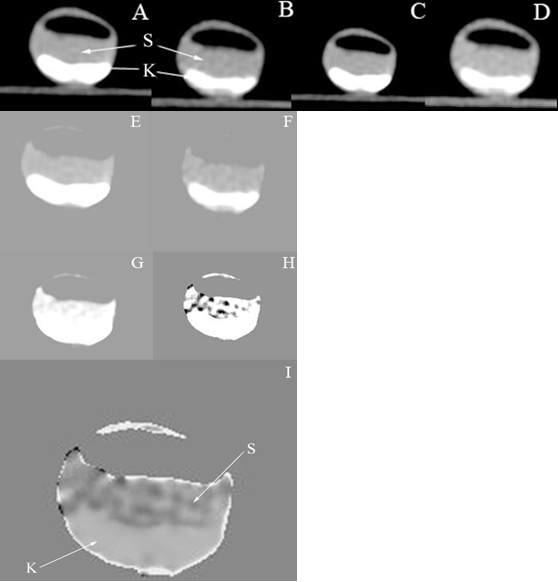


Dual-Energy index image generation

Cross section exemplary for a volumetric image stack. The improvement of differentiation within the non-calcified plaque (S) while maintaining detectability of calcification (K) is visible from E to I.

A: 70kV image

B: Sn150kV image

C: A+100HU (all voxels)

D: B+100HU (all voxels)

E: All voxels from C with HU<0 were set to 0

F: All voxels from D with HU<0 were set to 0

G: = E+F (addition of all voxels)

H: E–F (subtraction of all voxels)

I: H/G (division of all voxels)
