# Supplementary figures and images for: Ultra-high-resolution and dual-energy computed tomography of carotid artery plaques differentiate symptomatic and asymptomatic patients by novel volumetric analysis
Source: Interdiscip Cardiovasc Thorac Surg. 2025 Jun 30;40(7):ivaf158. doi: 10.1093/icvts/ivaf158 (PMC12270255; doi:10.1093/icvts/ivaf158)

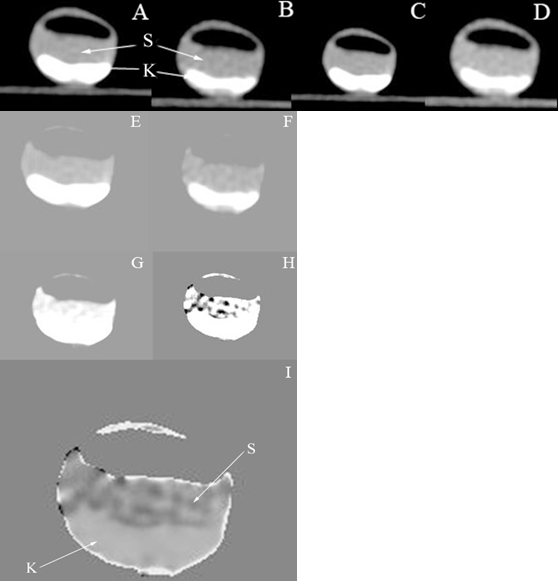

Supplement: ivaf158_Supplementary_Data [file ivaf158_supplementary_data.zip › supplementary figure 1.tif]
